# Alterations in gut microbiota and fecal metabolites in euthyroid autoimmune thyroiditis during early pregnancy

**DOI:** 10.1515/jtim-2026-0009

**Published:** 2026-04-04

**Authors:** Kan Chen, Zhenyu Lin, Yiyang Gao, Zhaoying Chen, Chenxi Zhang, Zhongyan Shan, Weiping Teng, Jing Li

**Affiliations:** Department of Endocrinology and Metabolism, The Institute of Endocrinology, NHC Key Laboratory of Diagnosis and Treatment of Thyroid Disease, The First Hospital of China Medical University, Shenyang, Liaoning Province, China; Department of Pulmonary and Critical Care Medicine, The Fourth Hospital of China Medical University, Shenyang, Liaoning Province, China

**Keywords:** autoimmune thyroiditis, adverse pregnancy outcomes, gut microbiota, fecal metabolites, 16S rDNA sequencing

## Abstract

**Background and Objectives:**

Euthyroid autoimmune thyroiditis (AIT) during early pregnancy has been linked to adverse pregnancy outcomes, yet the underlying mechanisms remain unclear. This study aimed to investigate the differences in gut microbiota (GM) composition and fecal metabolites between patients with euthyroid AIT and healthy women in the first trimester of pregnancy, and to explore potential associations between them.

**Methods:**

A total of 26 pregnant women with euthyroid AIT and 30 healthy pregnant women in their first trimester were enrolled. Gut microbiota profiles were analyzed using 16S rDNA gene sequencing, while fecal metabolomic profiling was performed *via* ultrahigh-performance liquid chromatography-mass spectrometry. Correlations between GM and fecal metabolites were further evaluated.

**Results:**

Significant diferences in GM composition were observed between euthyroid AIT patients and healthy controls. Metabolomic analysis revealed distinct fecal metabolic signatures in euthyroid AIT patients. Kyoto Encyclopedia of Genes and Genomes enrichment analysis indicated that diferential metabolites were mainly involved in arachidonic acid metabolism, alpha-linolenic acid metabolism, serotonergic synapses, and bile secretion pathways. Furthermore, a relationship between specific gut microbes and altered fecal metabolites was identified in the euthyroid AIT group.

**Conclusions:**

Pregnant women with euthyroid AIT in the first trimester exhibit distinct alterations in gut microbiota and fecal metabolic profiles, which may contribute to adverse pregnancy outcomes. Elucidating the correlations between GM and fecal metabolites may provide new insights into potential strategies for preventing and managing complications associated with euthyroid AIT in early pregnancy.

## Introduction

Autoimmune thyroiditis (AIT) is the most prevalent organ-specific autoimmune disorder, characterized by the presence of an autoinflammatory response in the thyroid gland accompanied by lymphocyte infiltration into the thyroid follicles.^[1]^ Primary serological manifestations of AIT include thyroid-stimulating hormone receptor antibodies (TRAb), thyroid peroxidase antibodies (TPOAb), and thyroglobulin antibodies (TgAb). Although TRAb are directly involved in Graves’ disease (GD) progression, the precise functions of TPOAb and TgAb remain uncertain. In pregnant women, TPOAb positivity varies between 5%–14%, and TgAb incidence ranges between 3%–18%.^[2]^ Thyroid autoimmunity has been associated with various adverse outcomes during pregnancy, such as miscarriage and preterm birth. Women with AIT exhibit an increased likelihood of infertility, miscarriage, and preterm birth, with AIT also contributing to postpartum thyroid dysfunction.^[3]^ Despite these findings, the pathogenesis of AIT is not yet fully comprehended. Current research suggests that AIT results from an immune abnormality triggered by environmental and other factors within the context of genetic susceptibility.^[4]^

Interest in the gut microbiota (GM) has increased considerably recently, with an increasing number of studies highlighting its association with disease. This association can partly be attributed to the influence of GM on systemic immune reactions.^[5, 6, 7]^ For instance, innate lymphocytes play a vital role in maintaining the GM equilibrium and controlling resistance against colonization by pathogens.^[8]^ Additionally, adaptive immune cells contribute to disease development by regulating the GM composition.^[9]^ Autoimmune disorders and the influence of the GM on host immune system are closely related.^[10, 11, 12]^ Although research on the relationship between GM and thyroid autoimmunity is lacking, GM and fecal metabolites have been suggested to influence thyroid immune responses, either directly or indirectly, potentially leading to the onset of autoimmune thyroid diseases (AITD).^[4,13]^ This viewpoint is consistent with our preliminary findings that were reported at the 2023 American Thyroid Association Annual and Centennial Celebration Meeting. At the meeting, we conducted an initial investigation into the GM composition and fecal metabolite profiles of patients with euthyroid AIT during the first trimester, and these findings guide this study.^[14]^ Furthermore, Su *et al*.^[15]^ found alterations in GM composition and metabolite profiles in patients with GD. Notably, the bacteria responsible for producing short-chain fatty acids (SCFAs) declined substantially, which corresponded with a decrease in the amount of SCFAs. As the anatomy, physiology, and immune functions of women change substantially during pregnancy, their GM is also affected.^[16, 17, 18]^ Owing to the potential AIT-associated adverse pregnancy outcomes, evaluating alterations in GM composition and their metabolites in patients with euthyroid AIT during the first trimester is warranted.

The purpose of this study was to examine the alterations in the GM composition and metabolite profiles of patients with euthyroid AIT in the first trimester. Moreover, we aimed to explore the potential relationships between these factors. Such findings could provide a clinical foundation for understanding the correlation between euthyroid AIT and changes in GM composition and metabolite profiles and offer potential avenues for preventing and managing euthyroid AIT-associate adverse pregnancy outcomes.

## Materials and methods

### Participant recruitment criteria

This study was based on the Northeast Regional Maternal-Fetal-Child-Adolescent Cohort. All participants were recruited at the First Hospital of the China Medical University Obstetrics Clinic between September 2018 and December 2019. Prior to the initiation of the study, an a priori power analysis was performed using GPower software (version 3.1.9.7). Assuming a large effect size (Cohen’s d = 0.8), α = 0.05, and power = 0.80, the required sample size was calculated to be 26 participants per group (total *N* = 52). In addition, the MetSizeR package was applied to estimate the required sample size for untargeted fecal metabolomics.^[19,20]^ Based on spectral bin counts, a false discovery rate (FDR) threshold of 0.05, and a probabilistic principal component analysis (PPCA) model, the minimum sample size was estimated to be 17 participants per group (total *N* = 34) (Supplementary Figure S1). Based on these calculations, a total of 26 euthyroid pregnant women in their first trimester with elevated levels of TPOAb (> 34 IU/mL), TgAb (> 115 IU/mL), or both were included in the AIT group.^[21]^ Thirty euthyroid pregnant women in their first trimester were selected as controls (CON) based on age and body mass index (BMI). According to previously-established gestation-specific reference intervals,^[22]^ euthyroid status during the first trimester was defined as FT4 13.35–19.01 pmol/L (2.5^th^–97.5^th^) and thyroid-stimulating hormone (TSH) 0.35–4.13 mIU/ L (2.5^th^–97.5^th^). The study’s inclusion criteria were as follows: Women aged 20–35 years, residing long-term in Northeast China, currently in their first trimester of pregnancy (7–13 weeks), and without prior thyroid treatments. Exclusion criteria were as follows: thyroid dysfunctions, > 13 weeks gestation, pre-pregnancy BMI outside the 18.0–28.0 kg/m^2^ range, histories of smoking, alcohol consumption, gastrointestinal diseases, or other chronic inflammatory conditions. The Medical Ethics Committee of the First Hospital of China Medical University approved this study (application number: 2018–22–3), and all participants provided written informed consent before sample collection.

### Sample collection and storage

Except for the serum samples used to test fasting blood glucose (FBG), blood lipids, and blood uric acid (BUA), samples were preserved at –80 °C until later use. Fecal samples were immersed in stool DNA stabilizer within collection tubes and immediately stored at –20 °C until future use.

### Biochemical assays

FBG levels were determined using the hexokinase approach. The cholesterol oxidase technique was used to measure total cholesterol (TC) and triglyceride (TG). Enzymatic colorimetric methods were used to evaluate high-density lipoprotein cholesterol (HDL-C), low-density lipoprotein cholesterol (LDL-C), and BUA levels. The analysis of thyroid function, including serum levels of TSH, free thyroxine (FT4), free triiodothyronine (FT3), TPOAb, and TgAb, was performed by a Roche E601 fully automated electrochemiluminescence immunoanalyzer.

### 16S rDNA gene sequencing

Genomic DNA was extracted using a NanoDrop 2000 UV microspectrophotometer (Thermo Fisher Scientific). The content and purity of the extracted DNA were evaluated using 1% agarose gel electrophoresis. The primers 341F (5’-CCTACGGGRSGCAGCAG-3’) and 806R (5’-GGACTACVVVGGGTATCTAATC-3’), attached to sequencing adapters, were used for Illumina Miseq PE250 sequencing. The PCR products were assessed, purified after amplification, and verified using 2% agarose gel electrophoresis. After quality control (QC), libraries were quantified using a fluorometer (Qubit@ 2.0) and an Agilent Bioanalyzer 2100 system. Sequencing was performed using an Illumina MiSeq PE250 platform after sample pooling. Taxonomic assignment based on the V3-V4 region was reliable up to the genus and family levels, whereas species-level resolution was not obtained. Therefore, subsequent analyses and interpretations were restricted to the genus or family levels.

### Liquid chromatography-tandem mass spectrometry (LC-MS/MS) analysis

QC samples were generated by combining portions of supernatant from each sample. The solution was then injected into an LC-MS/MS system. Separation was performed on a Hypesil Gold C18 column at a flow rate of 0.2 mL/min. The mobile phase in the positive mode comprised 0.1% formic acid (A) and methanol (B), whereas in the negative mode, it comprised 5 mmol/L ammonium acetate (pH 9.0, A) and methanol (B). Metabolite elution was performed using a gradient consisting of different proportions of mobile phase B at specific time intervals: 0–1.5 min with 2% mobile phase B, 1.6–14 min with 100% mobile phase B, and 14.1–17 min with 2% mobile phase B.

### Bioinformatical analysis

16S rRNA gene sequences were grouped into operational taxonomic units (OTUs) using Usearch with a similarity threshold of 0.97.^[23]^ Chimeras were filtered to obtain representative OTUs for species classification. The alpha diversity index of the samples was computed using the QIIME (V1.9.1) tool.^[24]^ The rank sum test was used to identify significant differences in alpha diversity indices. The QIIME (V1.9.1) tool was used to conduct a beta diversity analysis, which captured variations in species diversity between samples. Principal coordinate analysis (PCoA) revealed species diversity differences between samples. Linear discriminant analysis (LDA) effect size (LEfSe)^[25]^ assessed the impact of species abundance on differential effects, indicating that the community or species significantly influences sample classification.

For metabolomic analysis, we confirmed all putative identities by comparing the molecular mass data (m/z) of the samples with data from the KEGG, HMDB, and LIPIDMaps databases. MetaX,^[26]^ a comprehensive software program designed explicitly for metabolomic data management, was used for metabolite analysis, encompassing principal component analysis (PCA) and partial least squares discriminant analysis (PLS-DA). To determine statistical significance (*P*-value), we performed univariate analysis (*t*-test) and identified metabolites with significant differential expression based on criteria such as variable importance in projection (VIP) > 1.0, fold change (FC) > 1.5, FC < 0.667, and *P* < 0.05. Volcano plots generated using the R programming language and ggplot2 package were used to visualize the metabolite data based on log2 (FC) and log10 (*P*-value), enabling relevant metabolite identification. We conducted metabolic pathway enrichment analysis using MetaboAnalyst 3.0 to determine the most relevant metabolic pathways involved in the AIT group. The most significant pathways were identified by comparing the outcomes of the KEGG pathways.

### Statistical analysis

Data adhering to a normal distribution are presented as mean ± standard deviation. Group comparisons were conducted using Student’s t-test. Non-normally distributed data were represented using the median (interquartile range), and group comparisons were performed using the Wilcoxon–Mann–Whitney *U* test. For categorical variables, Fisher’s exact test was applied. Pearson’s correlation analysis was applied to the data fitting a bivariate normal distribution, whereas Spearman’s correlation analysis was used for other datasets. Given the relatively limited number of differential metabolites and the exploratory purpose of these analyses, multiple comparison correction was not applied, and the correlation results should be interpreted with caution. Statistical calculations were performed using SPSS 27.0, and *P* < 0.05 was considered statistically significant.

## Results

### Research participant characteristics

The evaluation of both groups (AIT and CON) showed no notable differences in age, BMI, FT3, FT4, TSH, FBG, BUA, TC, HDL-C, and LDL-C. However, TPOAb and TgAb levels significantly increased in the AIT group compared with those in the CON group (*P* < 0.01) (Table 1).

**Table 1:** General characteristics of all study participants

Variable	AIT group (*n* = 26)	CON group (*n* = 30)	*P*-value
BMI (kg/m^2^)	21.67 ± 2.72	21.22 ± 2.39	0.803
Age (years)	29.48 ± 3.38	30.21 ± 2.29	0.317
FT3 (pmol/L)	4.76 ± 0.62	5.01 ± 0.94	0.265
FT4 (pmol/L)	17.33 ± 2.13	16.41 ± 2.29	0.128
TSH (mIU/L)	1.89 (1.17 - 2.86)	1.57 (0.89 - 2.26)	0.175
TPOAb (IU/mL)	230.65 (48.24 - 360.95)	12.54 (8.72 - 15.72)	<0.01
TgAb (IU/mL)	372.10 (115.84 - 528.80)	10.76 (10.00 - 13.15)	<0.01
FBG (mmol/L)	5.01 ± 0.46	4.87 ± 0.34	0.171
BUA (mmol/L)	229.21 ± 46.61	207.67 ± 35.13	0.678
TG (mmol/L)	1.32 ± 0.70	1.19 ± 0.51	0.416
TC (mmol/L)	4.19 ± 0.63	4.21 ± 0.59	0.871
HDL-C (mmol/L)	1.71 ± 0.35	1.75 ± 0.37	0.651
LDL-C (mmol/L)	2.33 ± 0.67	2.42 ± 0.51	0.628

Data are presented as mean ± SD or median (interquartile range). The Student’s t test was used for normally distributed variables, and the Mann-Whitney U test was applied for non-normally distributed variables. FT4: free thyroxine; FT3: free triiodothyronine; TSH: thyroid-stimulating hormone; TPOAb: thyroid peroxidase antibody; TgAb: thyroglobulin antibody; FBG: fasting blood glucose; BUA: blood lipids, and blood uric acid; TG: triglyceride; TC: total cholesterol; HDL-C: high-density lipoprotein cholesterol; LDL-C: low-density lipoprotein cholesterol; BMI: body mass index.

### Pregnancy outcome-related clinical indicators

Pregnancy outcome-related clinical indicators were further compared between the two groups (Table 2). No significant differences were observed between the AIT and CON groups in gestational age at delivery, birth length, postpartum hemorrhage, preterm delivery, premature rupture of membranes (PROM), nuchal cord, fetal distress, and neonatal asphyxia. However, neonatal birth weight was significantly lower in the AIT group compared with the CON group (3240.38 ± 464.18 g *vs*. 3584.33 ± 448.23 g, *P* = 0.007). In addition, anemia before delivery was observed only in the AIT group (3/23 *vs*. 0/30), suggesting an increased trend in prevalence (*P* = 0.094).

**Table 2:** Pregnancy outcome-related clinical indicators of euthyroid AIT patients compared with CON in early pregnancy

Variable	AIT group	CON group	*P*-value
Gestational age at delivery (wk)	39.75 ± 1.10 (*n* = 26)	38.73 ± 4.50 (*n* = 30)	0.266
Birth weight (g)	3240.38 ± 464.18 (*n* = 26)	3584.33 ± 448.23 (*n* = 30)	0.007
Birth length (cm)	51.92 ± 3.59 (*n* = 26)	51.68 ± 2.74 (*n* = 28)	0.781
Anemia before delivery (*n*)	3 / 23	0 / 30	0.094
Postpartum hemorrhage (*n*)	3 / 25	0 / 24	0.235
Preterm delivery (*n*)	0 / 25	1 / 23	0.479
PROM (*n*)	1 / 24	0 / 24	1.000
Nuchal cord (*n*)	2 / 24	0 / 23	0.489
Fetal distress (*n*)	1 / 24	1 / 23	1.000
Neonatal asphyxia (*n*)	0 / 24	0 / 23	—

Comparisons between the two groups were performed using Fisher’s exact test. A difference was considered statistically significant if *P* < 0.05. Anemia before delivery referred to a hemoglobin concentration below 110 g/L within 1 week before delivery. AIT: autoimmune thyroiditis; CON: controls; PROM: premature rupture of membranes.

### Differences in the GM composition between AIT and CON during the first trimester

**Figure 1:**
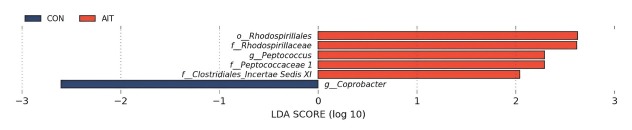
GM characteristics between the AIT and CON groups. (A) Species accumulation curves. The abscissa represents the number of sequenced samples, and the ordinate represents the detected OTUs. (B) Venn diagram of the AIT and CON groups. (C-G) The GM alpha diversity comparison between the AIT and CON groups, including observed_species, chao1, shannon, Simpson, and PD_whole_tree diversity indices. (H) GM beta diversity comparison between AIT and CON groups. Taxonomic proportions according to composition at the (I) phylum level and (J) genus level in the AIT and CON groups. GM: gut microbiota; AIT: autoimmune thyroiditis; CON: control; OTUs: operational taxonomic units.

High-throughput sequencing and QC of sequence extraction from 56 fecal samples yielded 3, 372, 065 high-quality sequences, with an average of 60, 215 sequences per sample. The number of OTUs increased with the number of fecal samples and exhibited a positive correlation (Figure 1A). However, as the number of samples approached 50, the cumulative curve gradually stabilized, suggesting that the size of the fecal samples in this study was adequate. Clustering analysis using the USEARCH software with a 97% similarity criterion yielded 614 OTUs for subsequent species taxonomy analysis. Among the OTUs, 505 were shared between the two groups. In contrast, 68 and 41 OTUs were unique to the CON and AIT groups, respectively (Figure 1B). This suggests partial differences in GM composition between the two groups, although the majority of OTUs were shared.

Alpha diversity evaluates the uniformity and abundance of species within the microbiota, whereas beta diversity measures the diversity common to the microbiota across different ecological ranges. Our initial focus examined alpha diversity within the GM of both groups. No notable differences were found between the groups in terms of metrics such as observed species, chao 1, Shannon, Simpson, and PD_whole_tree (Figure 1C-G). Beta-diversity analysis did not reveal significant separation between the AIT and CON groups (Figure 1H). Overall, both alpha- and beta-diversity analyses indicated that the gut microbial diversity was comparable between the two groups.

Considering the characteristics of 16S rDNA gene sequencing, we primarily presented the GM composition at the phylum and genus levels for both groups. At the phylum level, the majority of GM was composed of *Firmicutes* and *Bacteroidetes*, representing > 90% of the total GM (Figure 1I). At the genus level, the most abundant species were *Bacteroides*, *Prevotella, Faecalibacterium*, and *Roseburia*, constituting > 60% of the total sequences (Figure 1J).

**Figure 2:**
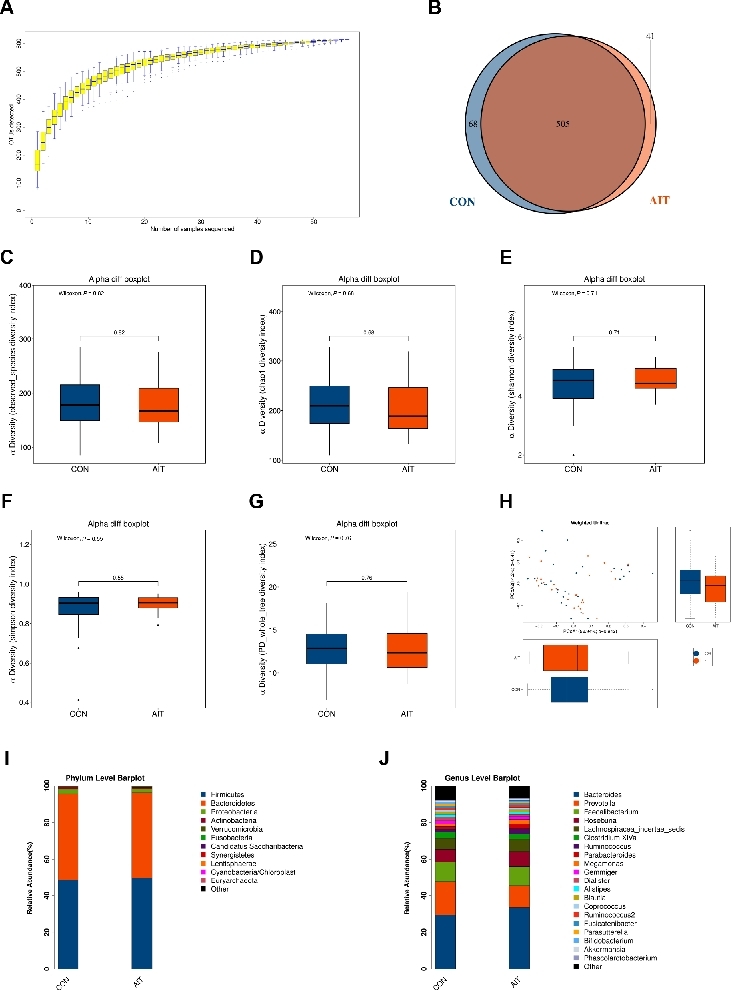
Linear discriminant analysis effect size (LEfSe) analysis. Histogram of LDA scores calculated for selected taxa showing significant differences in microbial type and abundance between AIT (orange) and control CON (dark blue) groups. The LDA score on the log10 scale is shown at the bottom. The significance of the microbial markers increased with an increase in the LDA score. LDA: linear discriminant analysis; AIT: autoimmune thyroiditis; CON: control.

LEfSe analysis was conducted to assess the extent to which the abundance of each component influenced the differences between the AIT and CON groups. Communities or species with significant contributions were identified and LDA scores were tallied for GM with significant differences. Six gut microbial biomarkers distinguished patients with AIT from the CON group at various taxonomic levels: the abundances of *o_Rhodospirillales*, *f_Rhodospirillaceae*, *g_Peptococcus*, *f_Peptococcaceae 1*, and *f_Clostridiales_Incertae Sedis XI* were notably higher in the AIT group than in the CON group (*P* < 0.05, LDA > 2), whereas *g_Coprobacter* showed higher enrichment in the CON group than in the AIT group (*P* < 0.05, LDA > 2) (Figure 2).

### Fecal metabolic differences between AIT and CON during the first trimester

**Figure 3:**
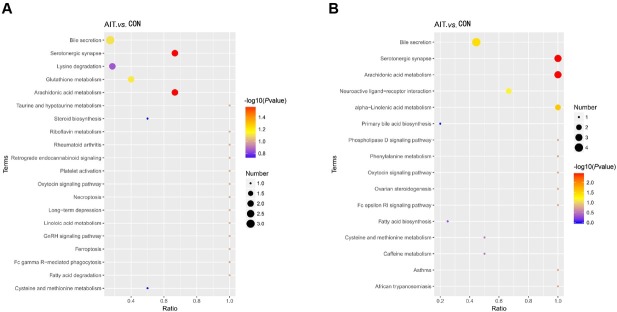
Score plots of the PCA and PLS-DA models and volcanic map of differential fecal metabolites between the AIT and CON groups. Score plot of PCA model in (A) positive-ion and (B) negative-ion mode. Score plot of the PLS-DA model in the (C) positive ion and (D) negative-ion modes. Volcanic map of differential fecal metabolites in the (E) positive ion and (F) negative-ion modes. The horizontal coordinate represents the fold change (log2 fold change) of fecal metabolites in different groups, and the vertical coordinate represents the significance level of the difference (-log10 *P*-value). Each point in the volcano plot represents a metabolite, with red dots indicating significantly upregulated expression, and green dots indicating significantly downregulated expression. The dot size corresponds to the Variable Importance in Projection (VIP) values. PCA: principal component analysis; PLS-DA: partial least squares-discriminant analysis; AIT: autoimmune thyroiditis; CON: control.

The metabolome profile depends on its susceptibility to external factors and rapid changes; therefore, QC sample correlation analysis is necessary to ensure the sample stability and precision. This analysis involved calculating the Pearson correlation coefficients for the QC samples. The results of this calculation showed R^2^ values close to 1 in the positive and negative ion modes (Supplementary Figure S2). This indicates robust detection and stable data quality. The PCA score plot suggested a trend of separation between the two groups, although some overlap was observed (Figure 3A and B). PLS-DA possesses superior classification capabilities compared to PCA. Therefore, the PLS-DA model was applied to further explore group separation and to assist in identifying differential fecal metabolites between the AIT and CON groups. The R2Y values surpassed the Q2Y values in both positive- and negative-ion modes, suggesting superior model quality. The PLS-DA model demonstrated clearer separation between the groups compared with PCA, indicating potential differences in metabolite profiles (Figure 3C and D).

In differential metabolite screening, we identified 706 fecal metabolites in the positive-ion mode and 355 fecal metabolites in the negative-ion mode. By establishing criteria with VIP values exceeding 1.0, and FC > 1.5 or < 0.667, we identified 89 differential metabolites in the positive-ion mode between the AIT and CON groups. Among these, 20 differential fecal metabolites were upregulated and 69 were downregulated in the AIT group compared to those in the CON group (Supplementary Table S1). In negative-ion mode, 46 differential metabolites were identified between the AIT and CON groups. Specifically, the AIT group demonstrated an increase in six fecal metabolites and a decrease in 40 fecal metabolites compared with the CON group (Supplementary Table S2). The volcano plot illustrates the variations in fecal metabolites between the AIT and CON groups (Figure 3E and F).

**Figure 4:**
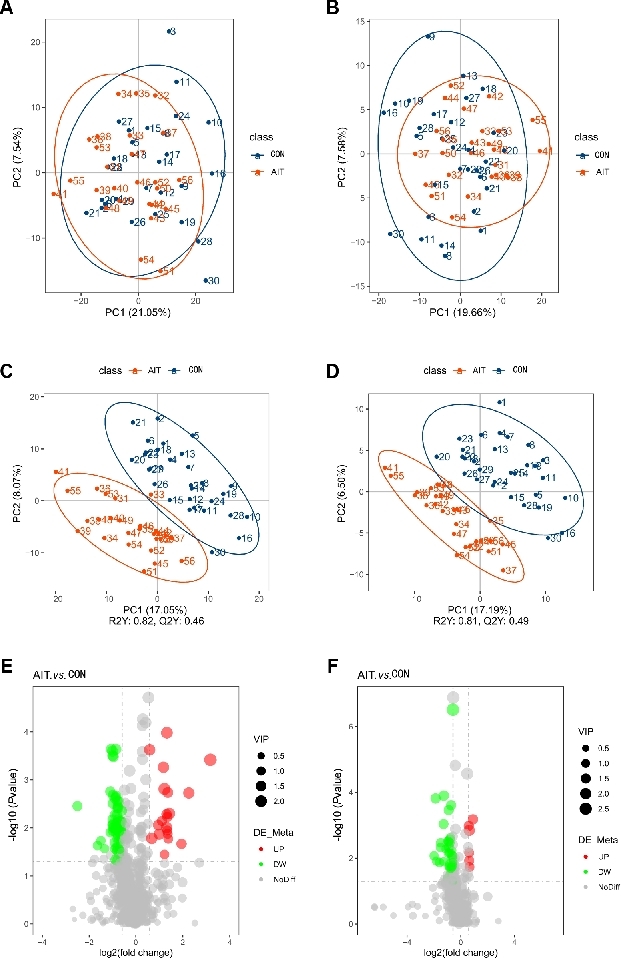
The KEGG enrichment analysis bubble plot between the AIT and CON groups. (A) KEGG enrichment analysis bubble plot in positive-ion mode showing that differential fecal metabolites were enriched in arachidonic acid metabolism and serotonergic synapse pathways. (B) The bubble plot of KEGG enrichment analysis in the negative-ion mode showing that differential fecal metabolites were enriched in pathways, including arachidonic acid metabolism, serotonergic synapse, alpha-linolenic acid metabolism, and bile secretion. The horizontal axis represents the ratio of the number of differential metabolites in the corresponding metabolic pathway to the total number of metabolites identified in that pathway. The greater the ratio, the greater the enrichment of differential metabolites in that pathway. The color of the dots represents the p-value from the hypergeometric test, with smaller values indicating better reliability and statistical significance. Dot size represents the number of differential metabolites in the corresponding pathway, with larger dots indicating a higher number of differential metabolites within the pathway. AIT: autoimmune thyroiditis; CON: control; KEGG: Kyoto Encyclopedia of Genes and Genome.

Given the large data volume and complexity in untargeted metabolomic analysis, further screening of differential fecal metabolites and pathway annotation using the KEGG database were required. These procedures facilitated the identification of key biochemical, metabolic, and signal transduction pathways, as well as differential metabolites. Ten distinct fecal metabolites were identified based on the KEGG enrichment analysis, primarily associated with four metabolic pathways: arachidonic acid (AA) metabolism, alpha-linolenic acid (ALA) metabolism, serotonergic synapses, and bile secretion (Figure 4). Some of these metabolites were involved in overlapping signaling pathways. Within the positive-ion mode, the AIT group showed reduced levels of AA and Thromboxane B2 in the AA metabolism and serotonergic synapse pathways compared to the CON group (Table 3). In the negative-ion mode, prostaglandin B2, F2alpha, and D2 exhibited decreased levels in these pathways in the AIT group compared to the CON group (Table 3). Additionally, traumatic acid and 13 (S)-HOTrE levels were reduced in the ALA metabolism pathway in the AIT group compared to those in the CON group (Table 3). In the bile secretion pathway, deoxycholic acid, Prostaglandin F2alpha, and chenodeoxycholic acid were downregulated, whereas uabain was increased in the AIT group compared to that in the CON group (Table 3).

**Table 3:** KEGG enrichment analysis results in both positive-ion and negative-ion modes

Mode	KEGG pathway	*P*-value	x	y	*n*	*N*	Enrich direct	Differential metabolites	Up/down
Pos	Arachidonic acid metabolism	0.026	2	3	14	142	over	Arachidonic acid	down
								Thromboxane B2	down
Pos	Serotonergic synapse	0.026	2	3	14	142	over	Arachidonic acid	down
								Thromboxane B2	down
Neg	Arachidonic acid metabolism	0.0036	3	3	13	79	over	Prostaglandin B2	down
								Prostaglandin F2alpha	down
								Prostaglandin D2	down
Neg	Serotonergic synapse	0.0036	3	3	13	79	over	Prostaglandin B2	down
								Prostaglandin F2alpha	down
								Prostaglandin D2	down
Neg	Alpha-Linolenic acid metabolism	0.025	2	2	13	79	over	Traumatic acid	down
								13(S)-HOTrE	down
Neg	Bile secretion	0.036	4	9	13	79	over	Deoxycholic acid	down
								Prostaglandin F2alpha	down
								Ouabain	up
								Chenodeoxycholic Acid	down

Pos: positive-ion mode; Neg: negative-ion mode; *N*: total number of metabolites involved in the KEGG pathway; *n*: number of differential metabolites among *N*; y: number of metabolites annotated to a specific KEGG pathway; x: number of differential metabolites enriched in this pathway; over: pathway significantly enriched in differential metabolites (x/*n* > y/*N*, *P* < 0.05); up: differential metabolite level increased in the AIT group compared with the CON group; down: differential metabolite level decreased in the AIT group compared with the CON group.

### Correlation analysis between differential GM and fecal metabolites

**Figure 5:**
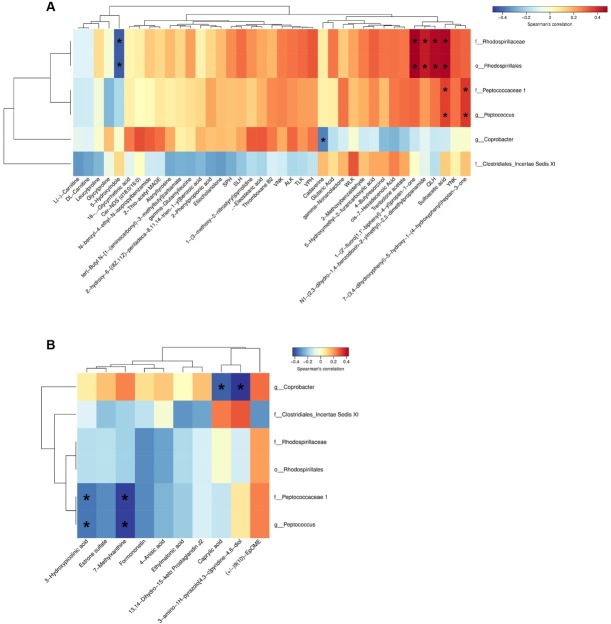
Correlation analysis between differential GM and fecal metabolites in the AIT group compared to the CON group. Correlation analysis heatmap between differential GM and fecal metabolites in (A) positive ion mode and (B) negative-ion mode. The horizontal and vertical coordinates represent differential GM and fecal metabolites, respectively, between the AIT and CON groups. The legend on the right side shows the correlation coefficients, where red and blue indicate a positive and negative correlation, respectively; ^*^P < 0.05. GM: gut microbiota; AIT: autoimmune thyroiditis; CON: control.

To investigate the interactions between distinct GM and fecal metabolites, we correlated differential GM obtained from 16S rDNA sequencing with differential fecal metabolites identified from metabolomics analysis using Spearman’s correlation analysis. Figure 5 demonstrates the association between these specific differential GM and fecal metabolites between the AIT and CON groups. In the positive-ion mode, an inverse relationship was identified between the abundance of *g_Coprobacter* and cadaverine levels. In contrast, a positive correlation was observed between the abundance of *g_Peptococcus*, sulfoacetic acid levels, and 7-(3, 4-dihydroxyphenyl)-5-hydroxy-1-(4-hydroxyphenyl) heptan-3-one (Figure 5A). In the negative-ion mode, *g_Coprobacter* abundance was negatively correlated with caprylic acid and 3-amino-1H-pyrazolo [4, 3-c] pyridine-4, 6-diol levels. In contrast, *g_Peptococcus* abundance was negatively correlated with levels of 7-Methylxanthine and 3-Hydroxypicolinic acid (Figure 5B).

### Correlations of altered gut microbiota and fecal metabolites with thyroid function

Correlation analyses were performed between the differential gut microbiota and fecal metabolites identified in the above analyses and thyroid function parameters (Table 4). Overall, most gut microbiota did not show significant correlations with thyroid function parameters. Only f_Rhodospirillaceae showed moderate positive correlations with FT4 (*r* = 0.344, *P* = 0.009), TPOAb (*r* = 0.342, *P* = 0.010), and TgAb (*r* = 0.329, *P* = 0.013).

**Table 4:** Spearman correlations between altered gut microbiota, fecal metabolites, and thyroid function parameters in early pregnancy

Category	Variable	FT3 (*r*, *P*)	FT4 (*r*, *P*)	TSH (*r*, *P*)	TPOAb (*r*, *P*)	TgAb (*r*, *P*)
Gut microbiota	*g_Bacteroides*	-0.009 (0.949)	-0.041 (0.763)	0.051 (0.710)	0.021 (0.877)	0.186 (0.169)
	*g_Bifidobacterium*	-0.119 (0.383)	-0.170 (0.211)	0.227 (0.092)	0.070 (0.610)	0.158 (0.245)
	*g_Coprobacter*	-0.107 (0.431)	0.101 (0.461)	-0.092 (0.502)	-0.171 (0.209)	-0.211 (0.119)
	*g_Prevotella*	0.067 (0.622)	0.049 (0.720)	-0.042 (0.758)	-0.097 (0.478)	-0.149 (0.273)
	*g_Lactobacillus*	0.254 (0.059)	0.096 (0.483)	-0.145 (0.287)	-0.088 (0.519)	0.079 (0.562)
	*g_Fusicatenibacter*	-0.110 (0.418)	-0.097 (0.479)	0.018 (0.895)	0.0002 (0.999)	0.087 (0.525)
	*g_Roseburia*	0.004 (0.975)	-0.173 (0.201)	0.148 (0.277)	0.067 (0.626)	0.154 (0.256)
	*g_Peptococcus*	-0.123 (0.365)	-0.060 (0.661)	0.070 (0.609)	0.142 (0.297)	0.188 (0.166)
	*g_Faecalibacterium*	-0.145 (0.285)	-0.097 (0.477)	-0.070 (0.610)	0.034 (0.804)	-0.055 (0.686)
	*f_Clostridiales_Incertae Sedis*					
	*XI*	-0.002 (0.986)	0.044 (0.750)	-0.076 (0.577)	-0.057 (0.675)	0.145 (0.285)
	*f_Rhodospirillaceae*	-0.231 (0.086)	0.344 (0.009)	-0.017 (0.901)	0.342 (0.010)	0.329 (0.013)
Fecal metabolites	Sulfoacetic acid	-0.147 (0.278)	0.262 (0.051)	0.027 (0.845)	0.623 (<0.001)	0.548 (<0.001)
	Cadaverine	-0.145 (0.287)	0.101 (0.459)	-0.084 (0.538)	0.156 (0.250)	0.278 (0.038)
	Arachidonic acid	-0.0004 (0.997)	-0.0001 (0.999402)	-0.245 (0.068)	-0.395 (0.003)	-0.398 (0.002)
	Thromboxane B2	-0.132 (0.332)	-0.096 (0.483)	-0.025 (0.857)	-0.223 (0.099)	-0.182 (0.179)
	7-Methylxanthine	0.207 (0.125)	-0.073 (0.592)	-0.109 (0.425)	-0.406 (0.002)	-0.394 (0.003)
	Prostaglandin F2α	-0.132 (0.334)	-0.204 (0.132)	-0.039 (0.776)	-0.416 (0.001)	-0.293 (0.028)
	Prostaglandin B2	0.055 (0.686)	-0.049 (0.720)	-0.103 (0.450)	-0.494 (<0.001)	-0.240 (0.074)
	3-amino-1H-pyrazolo [4,3-c]					
	pyridine-4,6-diol	0.105 (0.442)	-0.123 (0.368)	0.127 (0.351)	0.403 (0.002)	0.222 (0.100)
	Deoxycholic acid	0.006 (0.966)	-0.105 (0.443)	-0.235 (0.082)	-0.470 (<0.001)	-0.214 (0.114)
	Traumatic acid	-0.008 (0.955)	0.100 (0.465)	-0.028 (0.835)	-0.344 (0.009)	-0.077 (0.574)
	Caprylic acid	0.098 (0.473)	0.028 (0.838)	0.077 (0.575)	0.314 (0.018)	0.400 (0.002)
	13(S)-HOTrE	-0.165 (0.225)	-0.155 (0.254)	-0.076 (0.576)	-0.235 (0.081)	-0.276 (0.040)
	Ouabain	-0.071 (0.601)	0.176 (0.195)	-0.020 (0.884)	0.189 (0.162)	0.176 (0.195)
	Chenodeoxycholic acid	-0.209 (0.122)	-0.286 (0.033)	-0.041 (0.765)	-0.252 (0.061)	-0.264 (0.049)
	Prostaglandin D2	-0.151 (0.268)	-0.196 (0.147)	-0.185 (0.171)	-0.182 (0.180)	-0.043 (0.752)
	3-Hydroxypicolinic acid	0.160 (0.239)	-0.001 (0.994)	0.070 (0.610)	-0.165 (0.223)	-0.045 (0.741)

FT3: free triiodothyronine; FT4: free thyroxine; TSH: thyroid-stimulating hormone; TPOAb: thyroid peroxidase antibody; TgAb: thyroglobulin antibody. Data are Spearman’s rank correlation coefficients (*r*) with *P* values shown in parentheses. Correlations were assessed in the entire study cohort. Positive *r* indicates a direct correlation and negative *r* an inverse correlation. A difference was considered statistically significant if *P* < 0.05.

Compared with gut microbiota, several fecal metabolites exhibited more pronounced correlations with thyroid autoantibody levels. Sulfoacetic acid was strongly positively correlated with both TPOAb and TgAb (*r* = 0.623 and 0.548, *P* < 0.001), and cadaverine was positively correlated with TgAb (*r* = 0.278, *P* = 0.038). Several metabolites involved in AA-related pathways were negatively correlated with autoantibody levels, including AA, 7-methylxanthine, prostaglandin F2α, prostaglandin B2, and deoxycholic acid, with correlation coefficients ranging from-0.252 to-0.494 (*P* < 0.05). Among these, 7-methylxanthine was negatively correlated with both TPOAb (*r* =-0.406, *P* = 0.002) and TgAb (*r* =-0.394, *P* = 0.003). In addition, caprylic acid was positively correlated with TPOAb (*r* = 0.314, *P* = 0.018) and TgAb (*r* = 0.400, *P* = 0.002), whereas 13 (S)-HOTrE was negatively correlated with TgAb (*r* =-0.276, *P* = 0.040) and chenodeoxycholic acid was negatively correlated with FT4 (*r* =-0.286, *P* = 0.033) and TgAb (*r* =-0.264, *P* = 0.049).

To further evaluate whether these associations were independent of potential confounders, multivariable linear regression analyses were performed. Taking TPOAb or TgAb as the dependent variable, each differential metabolite was entered separately into a model, with simultaneous adjustment for f_Rhodospirillaceae, age at enrollment, and pre-pregnancy BMI (Supplementary Table S1). In these models, 3-amino-1H-pyrazolo [4, 3-c] pyridine-4, 6-diol remained positively associated with TPOAb, whereas 7-methylxanthine, prostaglandin B2, prostaglandin F2α, deoxycholic acid, and traumatic acid showed independent inverse associations with TPOAb. For TgAb, 7-methylxanthine, prostaglandin F2α, and chenodeoxycholic acid remained independently and inversely associated in the multivariable models, whereas caprylic acid and cadaverine remained positively associated with TgAb. At the pathway level, composite variables derived from AA-, ALA- and bile acid-related fecal metabolites were all inversely correlated with TPOAb, whereas only the AA-related composite showed a modest inverse correlation with TgAb (Supplementary Table S2).

## Discussion

**Figure 6:**
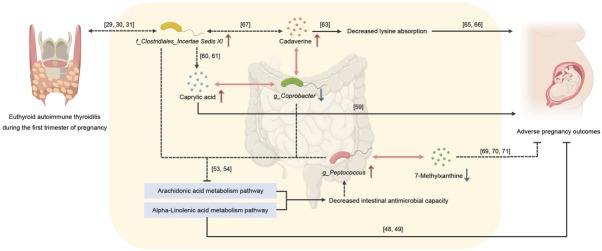
Association of altered GM and fecal metabolites with adverse pregnancy outcomes in euthyroid AIT during the first trimester of pregnancy. Solid connecting lines or arrows indicate the relevant mechanisms suggested based on literature reports or the results of this study, and dashed connecting lines or arrows indicate possible associations speculated based on relevant literature. Red and blue arrows indicate increases and depletions, respectively. GM: gut microbiota; AIT: autoimmune thyroiditis.

This study focused on examining the GM composition and its metabolomic profiles in patients with euthyroid AIT during the first trimester of pregnancy. Our research revealed notable disparities in the GM composition between patients with euthyroid AIT and healthy pregnant women in their first trimester. Additionally, patients with euthyroid AIT exhibited unique fecal metabolic characteristics, and specific GM were associated with differential stool metabolites. Notably, we observed a lower neonatal birth weight in the AIT group. Given that thyroid autoimmunity is associated with an increased risk of adverse pregnancy outcomes,^[3]^ we further explored whether the identified microbial and metabolic alterations were associated with the intensity of thyroid autoimmunity, which may provide biologically plausible links between gut dysbiosis and adverse pregnancy outcomes in euthyroid AIT. These findings may provide insights into the potential mechanisms underlying adverse pregnancy outcomes (Figure 6).

Peripheral thyroid homeostasis may be susceptible to alterations in GM, and the onset and progression of AITD may be influenced by variations in GM composition.^[27]^ Gong *et al*.^[28]^ found that AITD correlates with changes in GM at various taxonomic levels, including family, genus, and species. For instance, in the AITD cohort, beneficial microbes such as *Bifidobacterium* and *Lactobacillus* were diminished. In contrast, potentially harmful bacteria such as *Bacteroides fragilis* were markedly increased compared with healthy controls (HCs). Given the potential adverse outcomes of AIT in pregnancy,^[3]^ increasing attention has been paid to GM alterations in this population,^[29,30]^ and previous findings are summarized in Table 5. These findings suggest that GM alterations may provide a potential direction for exploring novel strategies in the management of TPOAb-positive SCH during the second and third trimesters, although causal relationships remain to be established. Our study found that patients with euthyroid AIT in their first trimester had a higher abundance of *f_Clostridiales_Incertae Sedis XI* and *g_Peptococcus*, whereas the abundance of *g_Coprobacter* was significantly lower than that in healthy pregnant women. Zhao *et al*.^[31]^ performed a cross-sectional analysis of 28 patients with Hashimoto’s thyroiditis (HT) and 16 HCs. Elevated *Clostridium_sensu_stricto1*, *Ruminococcustorques*, *Fusicatenibacter*, and other levels were found in HT patients. Gregoric *et al*.^[32]^ reported a patient with HT who repeatedly developed elevated serum TSH levels after receiving *Clostridium botulinum neurotoxin A* injections over ten years. Using bioinformatics techniques, they identified similarities between the amino acid sequences of *Clostridium botulinum neurotoxin A* and the thyroid autoantigen. This analysis revealed homologous epitopes shared between *Clostridium botulinum neurotoxin A* and thyroid autoantigens. Moreover, Benvenga *et al*.^[33]^ found that mycobacterial proteins of the GM, such as those produced by *Clostridium botulinum*, *Borrelia*, and *Yersinia*, may induce AIT because of the presence of similar antigenic determinants as thyroid peroxidase or thyroglobulin. The above results suggest that the elevated abundance of *f_Clostridiales Incertae Sedis XI* in the first trimester may be involved in the development of euthyroid AIT through mechanisms such as molecular mimicry; however, this association requires further confirmation in future studies.

**Table 5:** Summary of the studies on gut microbiota alterations in AITD patients during pregnancy

References	Year	Purpose	Subjects and grouping	The related main findings
(Wu, Yang, *et al*., 2022)^[29]^	2022	To investigate the differences in gut microbiota composition between TPOAb+ and TPOAb- SCH in T2.	75 TPOAb+ and 90 TPOAb- SCH in T2: (1) 12 TPOAb+ SCH without LT4 treatment; (2) 24 TPOAb+ SCH with low-dose LT4 treatment; (3) 39 TPOAb+ SCH with high-dose LT4 treatment; (4) 30 TPOAb- SCH without LT4 treatment; (5) 43 TPOAb- SCH with low-dose LT4 treatment; (6) 17 TPOAb- SCH with high-dose LT4 treatment.	(1) Firmicutes and Bacteroidetes were the dominant species, Faecalibacterium, Bacteroides, Prevotella 9, and others were the predominant genera in six groups. (2) Subdoligranulum was enriched in the gut microbiota of TPOAb+ SCH without LT4 treatment.
(Wu, Chi, *et al*., 2022)^[30]^	2022	To investigate the dynamics of gut microbiota from T2 to T3 in TPOAb+ and TPOAb- SCH.	64 TPOAb+ and 68 TPOAb- SCH in T2 and T3: (1) 8 TPOAb+ SCH without LT4 treatment in T2; (2) 8 TPOAb+ SCH without LT4 treatment in T3; (3) 56 TPOAb+ SCH with LT4 treatment in T2; (4) 56 TPOAb+ SCH with LT4 treatment in T3; (5) 18 TPOAb- SCH without LT4 treatment in T2; (6) 18 TPOAb- SCH without LT4 treatment in T3; (7) 50 TPOAb- SCH with LT4 treatment in T2; (8) 50 TPOAb- SCH with LT4 treatment in T3.	(1) Prevotella was enriched in T2 compared to T3, and Blautia and Agathobacter were increased in T3 compared to T2 in the gut microbiota of TPOAb+ SCH without LT4 treatment. (2) Faecalibacterium was enriched in T2 compared to T3, and Bifidobacterium was increased in T3 compared to T2 in the gut microbiota of TPOAb- SCH without LT4 treatment.

AITD, autoimmune thyroid disease; TPOAb, thyroid peroxidase antibodies; TPOAb+, TPOAb-positive; TPOAb-, TPOAb-negative; SCH, subclinical hypothyroidism; T2, the second trimester of pregnancy; T3, the third trimester of pregnancy; LT4, levothyroxine; low-dose LT4 ≤ 50 μg/d; high-dose LT4 > 50 μg/d.

Regarding metabolomics, several studies have reported associations between changes in metabolite concentrations and the development of AITD, and these changes may also be related to pregnancy outcomes, which requires further investigation.^[15,34,35,36,37]^ Alterations in GM ecology might influence the progression of GD through immune modulation, and metabolites have been proposed as potential intermediaries linking the GM and the thyroid, but additional evidence is needed to clarify these relationships.^[38]^ In this study, KEGG enrichment analysis revealed the inhibition of pathways such as AA and ALA metabolism. AA, a key membrane phospholipid, is released during inflammation.^[39]^ Free AA is metabolized *via* cyclooxygenase (COX), cytochrome P450 monooxygenase, and lipoxygenase (LOX) pathways to produce bioactive molecules such as prostaglandins (PGs) and leukotrienes.^[40]^ Long-chain polyunsaturated fatty acids (LCPUFAs), including AA, regulate immune function, and PGs can exert both pro- and anti-inflammatory effects.^[41,42]^ Omega-3 polyunsaturated fatty acids (PUFAs)-including ALA, eicosapentaenoic acid (EPA), and docosahexaenoic acid (DHA)-are anti-inflammatory lipids that must be obtained from diet or derived from ALA.^[43,44]^ Omega-3 PUFAs help maintain GM balance, whereas disproportionate intake may disrupt microbiota composition (*e.g*., increasing the *Firmicutes/Bacteroidetes* ratio).^[44,45]^ Omega-3 PUFAs support intestinal health by reducing oxidative stress, inhibiting the nuclear factor kappa-B pathway *via* peroxisome proliferator-activated receptor gamma (PPAR-γ), and preserving gut barrier integrity.^[46, 47, 48]^ Omega-3 PUFAs can also compete with AA for COX/LOX, reducing pro-inflammatory eicosanoids.^[49]^ Thus, the suppression of AA metabolism and ALA metabolic pathways, as well as imbalances in the expression levels of pro- and anti-inflammatory factors, could intensify inflammation and weaken the antimicrobial defenses of the gut. This could potentially result in heightened proliferation of detrimental bacteria, specifically *g_Peptococcus*. In addition to pathway-level differences, several fecal metabolites were associated with thyroid autoantibody levels. Specifically, sulfoacetic acid and cadaverine were positively correlated with thyroid autoantibodies, whereas 7-methylxanthine and multiple AA- and bile acid-related metabolites were inversely associated with thyroid autoantibodies. These findings suggest that altered fecal metabolic signatures may reflect an immunometabolic milieu linked to thyroid autoimmunity and may therefore be relevant to adverse pregnancy outcomes in euthyroid AIT. Long-term omega-3 intake has been reported to be associated with a reduced risk of thyroid autoimmunity and possibly postpartum thyroiditis,^[50]^ whereas insufficient omega-3 intake may be associated with adverse effects on neurodevelopment.^[51]^ DHA, the primary brain omega-3 PUFA, regulates neurotransmitters and brain function.^[52]^ Inhibition of the ALA pathway may reduce maternal-fetal DHA supply, which may raise concerns regarding potential risks of impaired offspring neurodevelopment in women with euthyroid AIT during pregnancy, and this association requires further prospective studies for validation.

The involvement of GM in the pathogenesis of various autoimmune diseases is linked to changes in fecal metabolite levels.^[53, 54, 55]^ Yang *et al*.^[53]^ reported increased *Escherichia-Shigella* abundance in primary Sjogren syndrome, positively correlated with four fecal metabolites. Vila *et al*.^[55]^ found in inflammatory bowel disease that GM composition influenced fecal metabolites more strongly than host lifestyle, genetics, or clinical phenotype. Moreover, previous studies have demonstrated a strong association between metabolite profiles and GM composition, even when various other influencing factors were considered. Altered GM composition likely causes metabolite changes, making these metabolites potential targets for microbiome-based therapies.^[56]^ Therefore, Pearson’s correlation analysis was applied to explore GM-metabolite relationships. While not proving causality, it revealed significant associations. Because categorical adverse pregnancy events were relatively infrequent in this cohort, examining thyroid autoimmunity as an intermediate host phenotype may help prioritize biologically plausible microbe-metabolite features potentially involved in the pathway linking gut perturbations to adverse pregnancy outcomes in euthyroid AIT.

In our study, *g_Coprobacter* abundance was negatively associated with caprylic acid and cadaverine levels in patients with euthyroid AIT during the first trimester. The genus *Coprobacter*, first identified in 2013,^[57]^ belongs to the *Barnesiellaceae* family within the *Bacteroidetes* phylum.^[58]^ Xiang *et al*.^[59]^ discovered a negative link between the abundance of *Coprobacter*, *Bacillales*, and *Lachnospira* and the risk of developing SLE. Gryaznova *et al*.^[60]^ found that patients with UC had significantly lower levels of *Coprobacter*, *Fusicatenibacter*, and *Butyricimonas* than the HCs. Caprylic acid (octanoic acid) is used in IVF protein supplements, but may adversely affect embryonic development, implantation, and fetal growth; thus, its use should be minimized.^[61]^ Furthermore, previous research indicated a positive association between the abundance of *Clostridium sensu stricto 1* and the levels of serum acetic acid and octanoic acid in patients with advanced chronic kidney disease taking oral AST-120.^[62]^ *Clostridium kluyveri* can transform ethanol and acetate into butyrate, caproate, and caprylate through a chain elongation mechanism.^[63]^ Thus, the increased abundance of *f_Clostridiales Incertae Sedis XI* may also be associated with elevated caprylic acid levels in patients with euthyroid AIT during the first trimester. Colonic microbiota ferment proteins to produce polyamines (*e.g*., spermine, spermidine, putrescine, cadaverine). Cadaverine is derived from L-lysine, an essential dietary amino acid important for growth and brain development; excess cadaverine may impair lysine utilization and cause adverse health effects.^[64, 65, 66, 67, 68]^ In addition, analysis of predicted metabolites from a colorectal cancer (CRC) dataset showed that cadaverine, creatine, and amino acids were enriched in CRC and positively correlated with related microorganisms such as *Clostridium symbiosum* and *Peptostreptococcus stomatis*.^[69]^ Therefore, the decreased abundance of g_Coprobacter and increased abundance of *f_Clostridiales_Incertae Sedis XI*, together with altered caprylic acid and cadaverine levels and their positive associations with thyroid autoantibodies, might contribute to the occurrence of adverse pregnancy outcomes in patients with euthyroid AIT during the first trimester. Nevertheless, whether these metabolites directly influence pregnancy outcomes or represent downstream correlates of gut-host interactions requires confirmation in longitudinal and mechanistic studies.

Our findings indicate a negative association between the abundance of *g_Peptococcus* and the levels of 7-Methylxanthine in patients with euthyroid AIT during the first trimester. 7-Methylxanthine, a derivative of 1, 3, 7-trimethylxanthine, is used medically (*e.g*., in eye health). Its derivative pentoxifylline shows anti-inflammatory, antioxidant, vasodilatory, and rheological effects, and may help prevent reperfusion injury.^[70,71]^ Pentoxifylline may also improve IVF outcomes (*e.g*., endometrial thickness, oocyte quality)^[72]^ and neonatal outcomes (*e.g*., higher birth weight, lower mortality) by reducing oxidative stress and inflammation.^[73]^ Therefore, the increased abundance of *g_Peptococcus* and decreased 7-Methylxanthine level might potentially be linked to the incidence of adverse pregnancy outcomes among patients with euthyroid AIT during the first trimester. In addition, the inverse association between 7-methylxanthine and thyroid autoantibodies observed in our cohort suggests that reduced 7-methylxanthine may align with a more pronounced autoimmune state, providing a potential pathway through which *g_Peptococcus*-related metabolic alterations could be linked to adverse pregnancy outcomes in euthyroid AIT.

This study has certain limitations. First, although the a priori power analysis indicated that the sample size was sufficient to detect large effect sizes, the overall number of participants remained limited, which may have reduced the ability to detect small or moderate effects, particularly given the high heterogeneity of microbiota and metabolomics data. In addition, a subset of participants had TSH levels > 2.5 mIU/L, but subgroup analyses based on this threshold could not be performed due to the limited sample size, which may have masked subtle associations. Moreover, because the number of differential metabolites identified was limited, stringent multiple testing correction was not applied, which may increase the risk of false positives; thus, these findings should be interpreted with caution. Second, although the AIT and CON groups were matched for age and BMI, other potential confounders (*e.g*., psychological stress and dietary habits) may still have influenced the results, thereby limiting the generalizability of our findings to populations with different genetic backgrounds and lifestyles. Finally, the functional significance of the differentially expressed GM and metabolites has not yet been validated *in vivo* or *in vitro*; therefore, the present findings are primarily associative and are insufficient to support definitive mechanistic inference. Future larger, multicenter studies incorporating mechanistic and functional validation experiments are warranted to confirm the causal roles of key microbes and metabolites and to further elucidate these associations with adverse pregnancy outcomes.

## Conclusions

In summary, we identified variations in the GM composition between patients with euthyroid AIT and healthy pregnant women during their first trimester. The decrease in the abundance of *g_Coprobacter* and the increase in the abundance of *f_Clostridiales_Incertae Sedis XI* and *g_Peptococcus* may be linked to adverse pregnancy outcomes among patients with euthyroid AIT during the first trimester. Patients with euthyroid AIT also exhibit unique fecal metabolic characteristics at this stage, including AA and ALA metabolic pathway inhibition. Additionally, a negative correlation was observed between *g_Coprobacter* abundance, caprylic acid, and cadaverine levels. Similarly, *g_Peptococcus* abundance was negatively correlation with 7-Methylxanthine level. Our study findings have the potential to enhance the understanding of the pathogenesis leading to adverse pregnancy outcomes in patients with euthyroid AIT during the first trimester. These insights offer new perspectives on the regulation of GM composition and fecal metabolic balance as potential preventive and therapeutic approaches for AIT-related adverse pregnancy outcomes.

## Supplementary Material

Supplementary Material Details
