# Supplementary material for: Alterations in gut microbiota and fecal metabolites in euthyroid autoimmune thyroiditis during early pregnancy
Source: J Transl Int Med. 2026 Apr 4;14(2):259–75. doi: 10.1515/jtim-2026-0009 (PMC13110464; doi:10.1515/jtim-2026-0009)
Supplement: Supplementary file 1 — Supplementary Material Details [file jtim-2026-0009_sm.pdf]

Supplementary materials

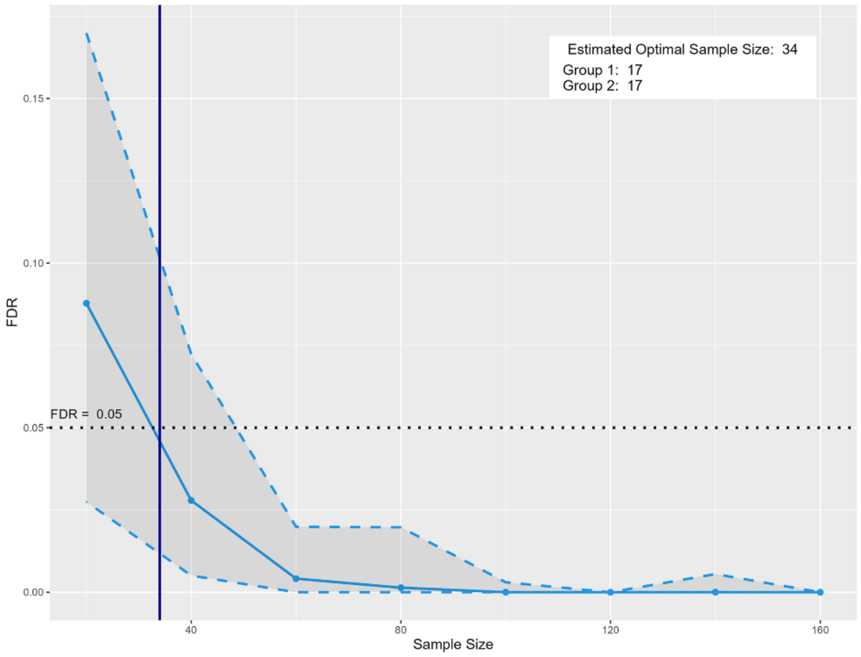

Supplementary Figure S1. Sample size estimation for untargeted fecal metabolomics using the MetSizeR package. Based on spectral bin counts, a FDR threshold of 0.05, and a PPCA model, the minimum required sample size was estimated as 17 participants per group (total *N* = 34).

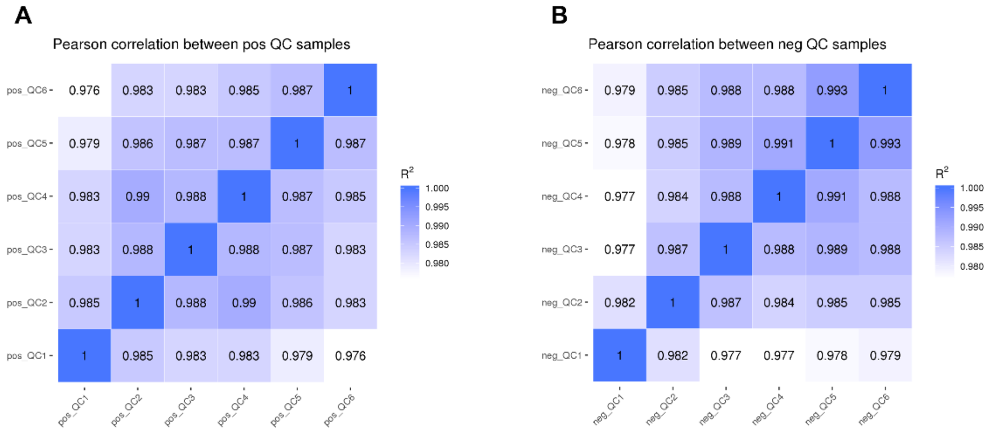

Supplementary Figure S2. Pearson correlation analysis of QC samples. (A) Pearson correlation among positive ion QC samples. (B) Pearson correlation among negative ion QC samples. A higher correlation of QC samples (R^2 values close to 1) indicates better stability of the assay process and higher data quality.

Supplementary Table S1. Multivariable linear regression models of differential gut microbiota and fecal metabolites with thyroid autoantibody levels in early pregnancy

**Thyroid autoantibody**	**Fecal metabolites**	**Predictor**	**β（per 1-SD increase）**	**95% CI**	***P*-value**	**Model R^2**
TPOAb	Sulfoacetic acid	Sulfoacetic acid	39.625	-4.547 to 83.797	0.085	0.091

		f_Rhodospirillaceae	3.97	-39.065 to 47.005	0.857	
		Age at enrollment	-34.578	-77.381 to 8.225	0.120	
		Prepregnancy BMI	16.936	-23.972 to 57.843	0.421	
	Arachidonic acid	Arachidonic acid	-13.057	-55.201 to 29.088	0.546	0.043
		f_Rhodospirillaceae	13.978	-28.455 to 56.412	0.521	
		Age at enrollment	-19.188	-63.143 to 24.768	0.396	
		Prepregnancy BMI	20.412	-21.363 to 62.187	0.343	
	7-Methylxanthine	7-Methylxanthine	-43.952	-83.917 to -3.986	0.036	0.117
		f_Rhodospirillaceae	8.875	-32.040 to 49.790	0.673	
		Age at enrollment	-12.443	-53.940 to 29.055	0.559	
		Prepregnancy BMI	20.715	-19.338 to 60.768	0.316	
	Prostaglandin F2α	Prostaglandin F2α	-49.097	-88.231 to -9.964	0.017	0.138
		f_Rhodospirillaceae	10.639	-29.511 to 50.790	0.606	
		Age at enrollment	-12.121	-52.916 to 28.675	0.563	
		Prepregnancy BMI	16.879	-22.830 to 56.589	0.409	
	Prostaglandin B2	Prostaglandin B2	-57.32	-97.195 to -17.445	0.007	0.166
		f_Rhodospirillaceae	5.2	-34.770 to 45.170	0.800	
		Age at enrollment	-12.483	-52.368 to 27.402	0.542	
		Prepregnancy BMI	3.174	-37.726 to 44.074	0.880	
	3-amino-1H-pyrazolo[4,3-c]pyridine-4,6-diol	3-amino-1H-pyrazolo[4,3-c]pyridine-4,6-diol	78.02	43.198 to 112.842	≤0.001	0.301
		f_Rhodospirillaceae	20.682	-15.384 to 56.749	0.266	
		Age at enrollment	-15.026	-51.088 to 21.036	0.418	
		Prepregnancy BMI	28.007	-7.755 to 63.770	0.131	
	Deoxycholic acid	Deoxycholic acid	-47.277	-86.964 to -7.591	0.024	0.129
		f_Rhodospirillaceae	9.655	-30.819 to 50.129	0.642	
		Age at enrollment	-22.385	-62.421 to 17.652	0.278	
		Prepregnancy BMI	9.27	-31.748 to 50.288	0.660	
	Traumatic acid	Traumatic acid	-43.646	-83.062 to -4.229	0.035	0.118
		f_Rhodospirillaceae	9.761	-31.013 to 50.536	0.641	
		Age at enrollment	-15.281	-56.213 to 25.651	0.468	
		Prepregnancy BMI	16.497	-23.761 to 56.755	0.426	
	Caprylic acid	Caprylic acid	21.552	-18.525 to 61.629	0.297	0.057
		f_Rhodospirillaceae	17.492	-24.457 to 59.441	0.418	
		Age at enrollment	-22.798	-64.470 to 18.875	0.289	
		Prepregnancy BMI	21.261	-20.126 to 62.647	0.319	
TgAb	Sulfoacetic acid	Sulfoacetic acid	21.329	-48.011 to 90.670	0.549	0.018
		f_Rhodospirillaceae	9.538	-58.018 to 77.094	0.783	
		Age at enrollment	-27.509	-94.702 to 39.683	0.426	
		Prepregnancy BMI	-1.588	-65.804 to 62.628	0.962	
	Cadaverine	Cadaverine	65.391	6.119 to 124.663	0.035	0.094
		f_Rhodospirillaceae	20.943	-41.098 to 82.984	0.511	
		Age at enrollment	-25.502	-87.274 to 36.269	0.422	
		Prepregnancy BMI	2.389	-58.878 to 63.655	0.939	
	Arachidonic acid	Arachidonic acid	-22.578	-86.982 to 41.826	0.495	0.02
		f_Rhodospirillaceae	12.979	-51.867 to 77.824	0.696	
		Age at enrollment	-14.399	-81.570 to 52.772	0.676	
		Prepregnancy BMI	-0.732	-64.571 to 63.107	0.982	

7-Methylxanthine	7-Methylxanthine	-83.068	-142.719 to -23.417	0.009	0.137
	f_Rhodospirillaceae	3.071	-57.998 to 64.139	0.922	
	Age at enrollment	-1	-62.937 to 60.937	0.975	
	Prepregnancy BMI	-0.297	-60.079 to 59.485	0.992	
Prostaglandin F2α	Prostaglandin F2alpha	-83.859	-142.840 to -24.878	0.007	0.142
	f_Rhodospirillaceae	7.31	-53.203 to 67.823	0.814	
	Age at enrollment	-2.414	-63.899 to 59.071	0.939	
	Prepregnancy BMI	-6.748	-66.597 to 53.100	0.826	
Caprylic acid	Caprylic acid	66.386	7.155 to 125.617	0.033	0.096
	f_Rhodospirillaceae	21.593	-40.405 to 83.591	0.498	
	Age at enrollment	-20.044	-81.633 to 41.545	0.526	
	Prepregnancy BMI	0.729	-60.437 to 61.895	0.981	
13(S)-HOTrE	13(S)-HOTrE	-38.873	-102.940 to 25.195	0.240	0.038
	f_Rhodospirillaceae	11.065	-53.173 to 75.304	0.737	
	Age at enrollment	-11.012	-76.830 to 54.806	0.744	
	Prepregnancy BMI	-8.345	-73.223 to 56.533	0.802	
Chenodeoxycholic Acid	Chenodeoxycholic Acid	-67.501	-127.670 to -7.332	0.032	0.097
	f_Rhodospirillaceae	13.857	-47.945 to 75.659	0.662	
	Age at enrollment	-19.98	-81.565 to 41.605	0.528	
	Prepregnancy BMI	-13.095	-75.488 to 49.297	0.683	

TPOAb: thyroid peroxidase antibody; TgAb: thyroglobulin antibody; BMI: body mass index. Data are presented as standardized regression coefficients (β per 1-SD increase) with 95% confidence intervals and *P* values. For each fecal metabolite, a separate linear regression model was fitted with TPOAb or TgAb as the dependent variable and f_Rhodospirillaceae, the metabolite of interest, age at enrollment, and pre-pregnancy BMI as independent variables. Model R^2 denotes the proportion of variance in the outcome explained by all covariates included in the corresponding model. Analyses were performed in the entire study cohort. Positive β indicates a direct association and negative β an inverse association. A difference was considered statistically significant if $P < 0.05$.

Supplementary Table S2. Spearman correlations between pathway-related fecal metabolites and thyroid autoantibody levels in early pregnancy

Metabolic pathway	Thyroid autoantibody	*r*	*P*-value
AA pathway	TPOAb	-0.471	<0.001
	TgAb	-0.274	0.041
ALA pathway	TPOAb	-0.418	0.001
	TgAb	-0.180	0.185
Bile acid-related pathway	TPOAb	-0.315	0.018
	TgAb	-0.180	0.184

TPOAb: thyroid peroxidase antibody; TgAb: thyroglobulin antibody. For each metabolic pathway, a composite variable was derived by standardizing metabolite intensities and averaging across metabolites assigned to that pathway. Correlations were

assessed in the entire study cohort. A difference was considered statistically significant if $P < 0.05$.
